# Genomic heterogeneity and lineage-specific beta-lactamases in recurrent *Achromobacter* bloodstream infection patients

**DOI:** 10.1080/22221751.2025.2547721

**Published:** 2025-08-26

**Authors:** Hsien-Po Huang, Yao-Ting Huang, Ting-Kuang Yeh, Yi-Pei Lee, Ting-Wei Lin, Hui-Hsien Pan, Po-Yu Liu

**Affiliations:** a Division of Infectious Diseases, Department of Internal Medicine, Taichung Veterans General Hospital, Taichung, Taiwan; b Department of Computer Science and Information Engineering, National Chung Cheng University, Chiayi, Taiwan; c Division of Infectious Diseases, Department of Pediatrics, Chung Shan Medical University Hospital, Taichung, Taiwan; d Division of Infectious Diseases, Department of Pediatrics, Taichung Veterans General Hospital, Taichung, Taiwan; e School of Medicine, National Yang Ming Chiao Tung University, Taipei, Taiwan; f Department of Post-Baccalaureate Medicine, College of Medicine, National Chung Hsing University, Taichung, Taiwan

**Keywords:** *Achromobacter*, bacteraemia, whole-genome sequencing, antimicrobial resistance, beta-lactamases

## Abstract

Recurrent *Achromobacter* infections pose significant clinical challenges due to antimicrobial resistance and within-host evolution. This study investigates the genetic and phenotypic changes among *Achromobacter* isolates using next-generation sequencing. We retrospectively analyzed 65 *Achromobacter* infection cases at a tertiary hospital in Taiwan from 2016 to 2023. Whole-genome sequencing of 12 isolates from patients with recurrent bloodstream infections was performed using Oxford Nanopore Technology. Resistance genes and beta-lactamases were identified, and genome similarity was assessed using average nucleotide identity (ANI) for phylogenetic analysis. Recurrent infections were significantly associated with bloodstream and urinary tract infections (p < 0.01). Whole-genome sequencing improved species identification over matrix-assisted laser desorption/ionization-time-of-flight mass spectrometry (MALDI-TOF MS), leading to the discovery of a novel *Achromobacter* species and the first identification of *A. insuavis* as a bacteraemia pathogen. Beta-lactamases grouped according to phylogenetic clades, indicating lineage-specific resistance patterns. Missense mutations in genes such as *siaT*, *rapA*, *atzEa1*, *AL523_09115*, and *clpA* correlated with changes in antimicrobial resistance profiles, suggesting in vivo evolution during recurrent infections. This study enhances understanding of *Achromobacter* genomic heterogeneity and underscores the importance of whole-genome sequencing for accurate species identification and resistance detection. The findings highlight the need for larger-scale studies to monitor emerging variants and assess their clinical impact.

## GRAPHICAL ABSTRACT

**Figure:**
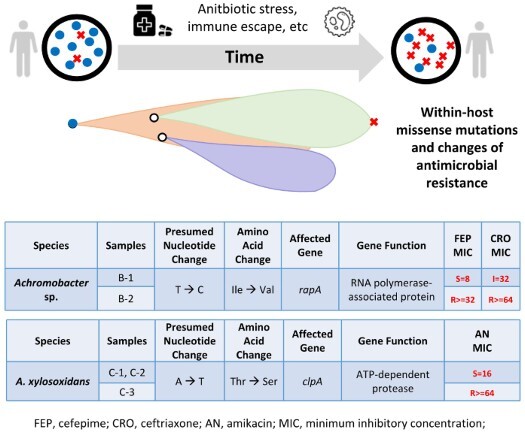

## Introduction

The genus *Achromobacter*, comprising over 20 officially recognized species [1], has emerged as an increasingly important opportunistic pathogen in clinical settings. While 15 species have been isolated from human samples (Supplementary Table 1), *Achromobacter xylosoxidans* remains the most prevalent, particularly in individuals with cystic fibrosis (CF), where it can establish chronic respiratory infections in Europe and the USA [2]. However, *Achromobacter* infections are also increasingly recognized in non-CF populations, primarily causing pneumonia and bacteraemia [3]. These infections can affect various other sites, including the skin, soft tissues, urinary tract, intra-abdominal organs, central nervous system, eyes, and ears [4]. Patients with medical devices like catheters and endotracheal tubes, underlying conditions such as diabetes mellitus, chronic renal failure, or heart diseases, and those with recent or past hospitalizations or healthcare exposures are particularly vulnerable [5], posing significant challenges for diagnosis and treatment.

The antibiotic resistance mechanisms of *Achromobacter* species are both diverse and concerning [2]. They include multidrug efflux pumps, constitutive chromosomal *OXA-114*-like β-lactamases, acquired extended-spectrum β-lactamases (ESBLs), *AmpC*-type β-lactamases, and metallo-β-lactamases (MBLs). This intrinsic and acquired resistance poses a formidable challenge to clinicians, as treatment options are often limited. Furthermore, numerous nonsynonymous single nucleotide polymorphisms (SNPs) have been identified, some affecting metabolic and virulence pathways, suggesting potential genomic adaptations during within-host microevolution [6]. This genomic plasticity suggests the potential for adaptation and within-host evolution during infection, potentially contributing to the development of antibiotic resistance and persistence within the host.

Despite the growing clinical importance of *Achromobacter* infections, our understanding of the genomic diversity and evolutionary dynamics of this genus remains incomplete. Traditional identification methods, such as biochemical tests and matrix-assisted laser desorption/ionization-time-of-flight mass spectrometry (MALDI-TOF MS), often lack the resolution to accurately differentiate between closely related species and identify emerging lineages. Moreover, the impact of within-host evolution on resistance development and clinical outcomes is not fully understood.

To address these knowledge gaps, our study aims to investigate the genetic and phenotypic variations among *Achromobacter* isolates. We detail the characteristics of recurrent *Achromobacter* infections and employ whole-genome sequencing (WGS) to achieve accurate species identification, characterize the resistome, and track within-host evolution through the analysis of longitudinal isolates. Our findings provide valuable insights into the genomic heterogeneity of *Achromobacter* species, the lineage-specific distribution of beta-lactamases, and the potential role of within-host evolution in the development of antibiotic resistance.

## Methods

### Study population

We conducted a retrospective observational study analyzing 65 cases of *Achromobacter* species infection in a tertiary hospital in Taiwan from 2016 to 2023 retrospectively. We reviewed each patient’s electrical chart by at least two authors and recorded each patient's culture results, characteristics, and antimicrobial susceptibility testing of each isolate. Inclusion criteria were patients with confirmed *Achromobacter* infection. Exclusion criteria included incomplete medical records or polymicrobial infections. Recurrent bloodstream infection (BSI) was defined as a positive blood culture obtained from a specimen collected 30 days or more after the initial infection. During the first treatment period, appropriate antibiotics were administered, and subsequent cultures showed negative results.

### Clinical microbiology laboratory methods

Cultures were identified using MALDI-TOF MS (BioMérieux). Antimicrobial susceptibility testing was performed by VITEK®2 (BioMérieux) and interpreted according to the Clinical and Laboratory Standards Institute (CLSI) breakpoints for “other non-Enterobacterales,” as specific breakpoints for *Achromobacter* species are not yet established.

### Genome sequencing and assembly

Longitudinal *Achromobacter* isolates obtained from bloodstream cultures of six patients with recurrent bacteraemia were selected for whole-genome sequencing. Genomic DNA was extracted using the Qiagen DNeasy blood and tissue kit (Qiagen Co., Germany). Libraries were prepared using the Rapid Barcoding Kit and sequenced using the Oxford Nanopore Technology (ONT) (GridION. R9.4 flowcells). Basecalling was performed using Guppy v6.3.4 with the SUP model. Reads were assembled de novo using Flye (https://github.com/fenderglass/Flye), then polished using the Racon (https://github.com/lbcb-sci/racon), the Medaka (https://github.com/nanoporetech/medaka), and the Homopolish (https://github.com/ythuang0522/homopolish).

### Resistome and phylogenetic analysis

The protein-coding genes, coding and non-coding RNAs in the chromosomes, and plasmids were annotated using the NCBI Prokaryotic Genome Annotation Pipeline (PGAP) [7]. Antibiotic-resistant genes (ARGs) were predicted by aligning protein-coding genes against the Comprehensive Antibiotic Resistance Database (CARD) [8]. Only ARGs with alignment coverage greater than 90% were retained. Efflux pumps were excluded from the ARG analysis. Beta-lactamases were separately predicted by using the curated hidden Markov models in NCBI AMRFinderPlus [9].

Pairwise average nucleotide identity (ANI) was calculated using FastANI [10] to assess genomic similarity between isolates and type strains of *A. xylosoxidans* (RM8376), *A. insuavis* (LMG26845), and *A. denitrificans* (FDAARGOS_786). ANI values were visualized using custom Python scripts. A 95% ANI threshold was used for species demarcation, consistent with current standards. Phylogenetic analysis was performed based on whole-genome sequences, including the aforementioned type strains, to determine the evolutionary relationships between isolates.

We also have undertaken a more detailed multi-locus sequence typing (MLST) analysis through PubMLST to improve the resolution of lineage relationships and better understand the evolutionary trajectories within *Achromobacter* species.

### Statistical assessment of within-host allele shifts

To identify SNP loci exhibiting significant allele frequency shifts between two recurrent-infected samples from the same patient, sequencing reads from each sample pair were aligned to a common reference genome derived from the initial isolate. Specifically, A-1 (CP150720) was used as the reference for A-1 and A-2; B-1 (CP150899) for B-1 and B-2; C-1 (CP150723) for comparisons of C-1 vs. C-2 and C-1 vs. C-3; and D-1 (CP150724) for D-1 vs. D-2. This approach ensured consistency in variant calling across recurrent episodes within each patient. For each variant locus, we determined the major allele per sample and retained only those loci where the major allele differed between the samples. We excluded non-coding regions and synonymous mutations to focus on non-synonymous, functional-relevant mutations.

To assess the statistical significance of allele frequency changes at each retained locus, we employed a Dirichlet-multinomial (DM) Likelihood Ratio test (LRT). This approach accounts for both multi-allelic counts and overdispersion commonly observed in high-throughput sequencing data, arising from sources such as amplification bias and sampling variability. Unlike the simple multinomial or binomial models (e.g. χ^2^, Fisher’s exact tests, negative-binomial test) that assume variance determined by sampling depth.

Under the null hypothesis H_0_, both samples are assumed to share a common underlying allele frequency vector *p* = (p_A_, p_C_, p_G_, p_T_) and a shared dispersion parameter φ. Under the alternative hypothesis (H_1_), the dispersion φ remains constant, but the two samples are allowed distinct allele frequency vectors, *p*^(1)^ and *p*^(2)^ such that *p*^(1)^ ≠ *p*^(2)^. We estimated model parameters by maximizing the log-likelihoods of the observed four-nucleotide allele counts under both hypotheses. Then, a likelihood ratio test (LRT), Λ = −2(ℓ_0_−ℓ_1_), whereas was used to quantify support for differential allele frequencies, where ℓ_0_ and ℓ_1_ represent the DM log-likelihoods under H_0_ and H_1_, respectively. This LRT thus provides a variance-aware test for detecting genuine allele-frequency shifts between samples.

### Ethics approval

The study was conducted in accordance with the guidelines of the Declaration of Helsinki and approved by the Institutional Review Board of Taichung Veterans General Hospital (CE24126B).

### Statistical analysis

Statistical analyzes were performed using SPSS v25.0 (IBM Corp., Armonk, NY). Fisher's exact test was used to compare recurrence rates among different infection sites. A *p*-value of < 0.05 was considered statistically significant.

## Results

### Characteristics of patients with recurrent Achromobacter infection

Among the 65 patients with *Achromobacter* infections, 23 (35.4%) had pneumonia, 18 (27.7%) had urinary tract infections (UTIs), 11 (16.9%) had bloodstream infections (BSIs), 8 (12.3%) had skin and soft tissue infections (SSTIs), and 3 (4.6%) had intra-abdominal infections (IAIs) (Supplementary Table 2). Recurrent infections occurred in 19 patients (29.2%). Notably, recurrence rates were significantly higher in patients with BSIs (54.5%, *p* = 0.007) and UTIs (44.4%, *p* = 0.007) compared to those with SSTIs (12.5%) and IAIs (33.3%) (Supplementary Table 2).

Of the six patients with recurrent bloodstream infections (Supplementary Table 3), four (66%) were female, and the median [IQR] age was 53 [39–61] years. Four (66%) patients had underlying malignancies. The suspected infection in four (66%) patients was related to central line infection, while the remaining two were attributed to recurrent cellulitis and vesico-rectal fistula infection, respectively. The median [IQR] interval between recurrences was 50 [42–69] days, and piperacillin-tazobactam was used as the definitive antibiotic in all cases.

### Improved and novel species identification by whole-genome sequencing

Whole-genome sequencing was performed on 13 *Achromobacter* isolates from the six patients with recurrent BSIs (see Methods). However, one isolate's sequencing yield of patient E was too low to assemble a complete genome, and therefore, the data was discarded. Comparison of species identification by MALDI-TOF MS and whole-genome sequencing revealed discrepancies in 5 out of 12 isolates (41.7%) (Table 1). Both methods identified A-2, C-1, C-2, C-3, D-2, and E-1 as *A. xylosoxidans*. However, isolates A-1 and D-1, initially identified to the genus level by MALDI-TOF MS, were confirmed as *A. xylosoxidans* through sequencing.

**Table 1.** Comparison of species identification by MALDI-TOF MS and whole-genome sequencing.

Samples ID	MALDI-TOF MS Identification	Whole-Genome Sequencing Identification
A-1	*Achromobacter* spp.	*A. xylosoxidans*
A-2	*A. xylosoxidans*	*A. xylosoxidans*
B-1	*A. denitrificans*	*Achromobacter* sp.
B-2	*Achromobacter* spp.	*Achromobacter* sp.
C-1	*A. xylosoxidans*	*A. xylosoxidans*
C-2	*A. xylosoxidans*	*A. xylosoxidans*
C-3	*A. xylosoxidans*	*A. xylosoxidans*
D-1	*Achromobacter* spp.	*A. xylosoxidans*
D-2	*A. xylosoxidans*	*A. xylosoxidans*
E-1	*A. xylosoxidans*	*A. xylosoxidans*
F-1	*Achromobacter* spp.	*A. insuavis*
F-2	*Achromobacter* spp.	*A. denitrificans*

**Figure 1.**
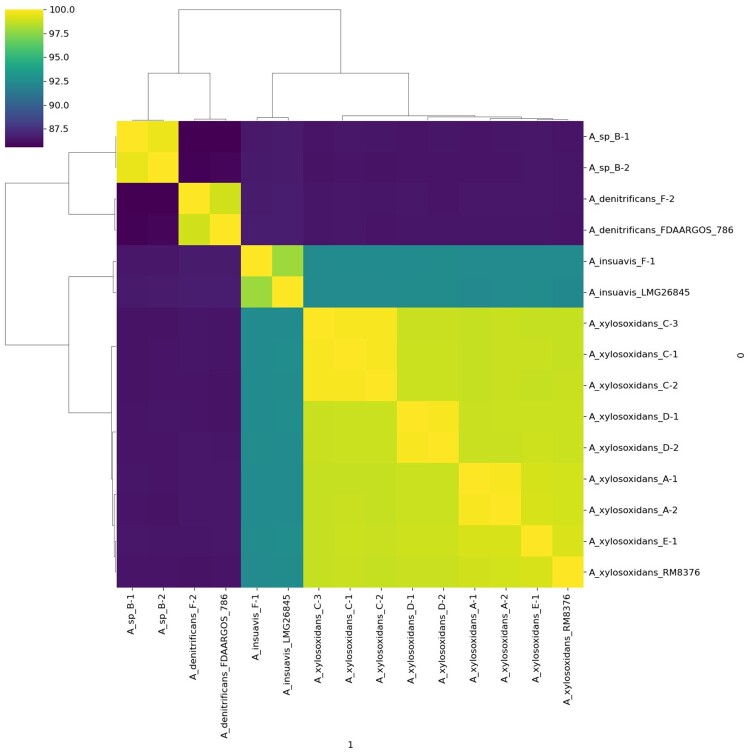
Heatmap of the average nucleotide identity (ANI) analysis and whole genome dendrogram of *Achromobacter* species. Isolates B-1 and B-2 exhibited a maximum ANI of 86% against all known *Achromobacter* type strains, falling below the 95% species demarcation threshold.

In Patient F, both isolates were identified as *Achromobacter* spp. by MALDI-TOF MS. Whole-genome sequencing revealed the first isolate to be *A. insuavis* and the second to be *A. denitrificans*, both with 99% ANI compared to their respective type strains (Figure 1). Importantly, WGS identified a novel *Achromobacter* species in patient B. Isolates B-1 and B-2 exhibited a maximum ANI of 86% against all known *Achromobacter* type strains, falling below the 95% species demarcation threshold. This finding suggests the presence of a previously uncharacterized lineage within the *Achromobacter* genus. Phylogenetic analysis based on WGS data revealed four distinct clades corresponding to the predicted species, highlighting the improved resolution offered by WGS compared to MALDI-TOF MS (Figure 1).

The MLST data has also been meticulously compiled (Supplementary Table 4). Our analysis revealed that each ANI-based cluster corresponds precisely to a specific sequence type (e.g. ST-315 for A-1 and A-2), demonstrating complete concordance between the ANI-based clustering and PubMLST typing. Additionally, the whole genome phylogeny of the 12 isolates with one outgroup (*A. piechaudii*) confirmed the same clustering of the ANI analysis (Supplementary Figure 1). By comparing a number of clinical and environmental *Achromobacter* strains, we observe the phylogenetic clusters tend to separate the pathogenic from the non-pathogenic strains and there are no significant clusters matching particular infection types (e.g. otitis, bloodstream infections) (Supplementary Figure 2).

### Within-host evolution of Achromobacter species during recurrent infection

**Figure 2.**
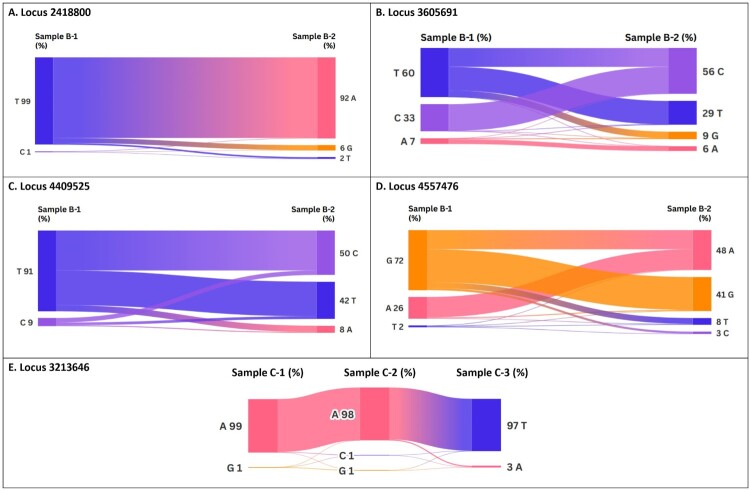
Sankey plot of single nucleotide polymorphism (SNP) of *Achromobacter* species strains in this study. Analysis of non-synonymous single nucleotide polymorphisms (SNPs) between sequential isolates from patients A, B, and C revealed a total of five missense mutations.

Analysis of non-synonymous single nucleotide polymorphisms (SNPs) between sequential isolates from patients A, B, and C revealed a total of five missense mutations (Table 2). These mutations affected genes involved in various cellular processes, including sialic acid transport (*siaT*), RNA polymerase function (*rapA*), cyanuric acid metabolism (*atzEa1*), peptide transport (*AL523_09115*), and stress response (*clpA*). For example, in Patient A, a mutation in the *siaT* gene resulted in a glutamine to leucine substitution at position 737 (Q737L) between isolates A-1 and A-2. Similarily, mutations T76S in *rapA*, I1636L in *atzEa1*, Q479A in *AL523_09115*, and G209D in *clpA* were observed in other isolates. These findings suggest the emergence of distinct sub-populations within the host during recurrent infection, potentially driven by adaptation to the host environment or antibiotic selection pressure (Figure 2).

**Table 2.** Identified missense mutations in sequential *Achromobacter* isolates. *siaT*, Sialic acid transporter; *rapA*, RNA polymerase-associated protein; *atzEa1*, 1-carboxybiuret hydrolase subunit *AtzE*; *AL523_09115*, Peptide ABC transporter; *clpA,* ATP-dependent *Clp* protease ATP-binding subunit.

Species	Samples	SNP locus	A	C	G	T	Nucleotide/Amino acid changes	Affected genes
*A. xylosoxidans*	A-1	2418800	0%	1%	0%	**99%**	T → A (Gln → Leu)	*siaT*
* *	A-2		92%	0%	6%	**2%**		
*Achromobacter* sp.	B-1	3605691	7%	33%	0%	**60%**	T → C (Ile → Val)	*rapA*
	B-2		6%	**56%**	9%	29%		
	B-1	4409525	0%	9%	0%	**91%**	T → C (Gln → Arg)	*atzEa1*
	B-2		8%	**50%**	0%	42%		
	B-1	4557476	26%	0%	**72%**	2%	G → A (Gly → Asp)	*AL523_09115*
* *	B-2		**48%**	3%	41%	8%		
*A. xylosoxidans*	C-1	3213646	**99%**	0%	1%	0%	A → T (Thr → Ser)	*clpA*
* *	C-2		**98%**	1%	1%	0%		
* *	C-3		3%	0%	0%	**97%**		

The bold font indicates the most likely nucleotide at each locus.

### Correlation of antimicrobial resistance and within-host missense mutations

Piperacillin-tazobactam, imipenem, and trimethoprim/sulfamethoxazole exhibited the highest in vitro activity against the *Achromobacter* isolates (Supplementary Table 5). Amikacin and gentamicin showed no activity against most isolates, except for one *A. xylosoxidans* isolate (sample C-2) and one *A. denitrificans* isolate (sample F-2). Cefepime, ceftriaxone, ciprofloxacin, and colistin demonstrated poor overall susceptibility. Moderate susceptibility was observed for tigecycline, ampicillin-sulbactam, and ceftazidime.

Isolate B-2 exhibited increased minimum inhibitory concentration (MICs) for cefepime, ceftriaxone, ciprofloxacin, and tigecycline compared to isolate B-1. This change coincided with the T76S mutation in *rapA*, suggesting a potential role for this mutation in the development of resistance. Similarly, isolate C-3 showed increased resistance to amikacin compared to isolates C-1 and C-2, which correlated with the G209D mutation in *clpA*.

### Lineage-specific distribution of beta-lactamases

Analysis of beta-lactamase genes revealed a clade-specific distribution of class A and D enzymes (Figure 1, Table 3). All *A. xylosoxidans* isolates harboured *bla*_OXA-114-like_ genes, with specific variants such as *bla*_OXA-114f_ in isolates A-1 and A-2, *bla*_OXA-114w_ in isolates C-1 to C-3, and *bla*_OXA-114j_ in isolates D-1 and D-2. The novel *Achromobacter sp.* isolates from Patient B possessed the *bla*_OXA-1238_ gene. In Patient F, *bla*_OXA-364_ was identified in the *A. insuavis* isolate (F-1), representing the first report of this gene in this species, while *bla*_AXC-2_ was found in the *A. denitrificans* isolate (F-2). The consistent presence of specific beta-lactamases within each clade suggests that these genes may be lineage-specific and contribute to intrinsic resistance profiles.

**Table 3.** Beta-lactamase genes and susceptibility identified in *Achromobacter* isolates.

Samples	GenBank accession number	Species	Class C beta-lactamase	Class D beta-lactamase	Class A beta-lactamase	Susceptibility to β-lactam
A-1	CP150720	*A. xylosoxidans*	*AmpC*	*OXA-114f*		Non-susceptible to cefepime and ceftriaxone
A-2	CP150721	*A. xylosoxidans*	*AmpC*	*OXA-114f*		Non-susceptible to cefepime and ceftriaxone
B-1	CP150899	*Achromobacter* sp.	*AmpC*	*OXA-1238*		Susceptible to cefepime, but non-susceptible to ceftriaxone
B-2	CP150897	*Achromobacter* sp.	*AmpC*	*OXA-1238*		Non-susceptible to cefepime and ceftriaxone
C-1	CP150723	*A. xylosoxidans*	*AmpC*	*OXA-114w*		Non-susceptible to cefepime and ceftriaxone
C-2	CP150722	*A. xylosoxidans*	*AmpC*	*OXA-114w*		Non-susceptible to cefepime and ceftriaxone
C-3	CP150898	*A. xylosoxidans*	*AmpC*	*OXA-114w*		Non-susceptible to cefepime and ceftriaxone
D-1	CP150724	*A. xylosoxidans*	*AmpC*	*OXA-114j*		Non-susceptible to cefepime and ceftriaxone
D-2	CP150719	*A. xylosoxidans*	*AmpC*	*OXA-114j*		Non-susceptible to cefepime and ceftriaxone
E-1	CP150718	*A. xylosoxidans*	*AmpC*	*OXA-114i*		Non-susceptible to cefepime and ceftriaxone
F-1	CP150896	*A. insuavis*	*AmpC*	*OXA-364*		Susceptible to cefepime, but non-susceptible to ceftriaxone
F-2	CP150725 CP150726	*A. denitrificans*			*AXC-2*	Susceptible to cefepime, but non-susceptible to ceftriaxone

## Discussion

Our study revealed that recurrent *Achromobacter* infections were predominantly associated with bloodstream infections (BSIs) and urinary tract infections (UTIs), consistent with previous research highlighting the predilection of this genus for these sites [3]. The characteristics of our BSI patients aligned with prior reports, showing a predominance of *A. xylosoxidans* infections in individuals with underlying malignancies and central venous catheter implants [11]. However, the significantly higher recurrence rates observed in BSIs and UTIs compared to other infection types underscore the need for heightened vigilance and targeted interventions in managing these cases.

Whole-genome sequencing proved more effective than MALDI-TOF MS for species identification, correcting discrepancies in 41.7% of isolates. The identification of a novel *Achromobacter* species and the first report of *A. insuavis* as a bacteraemia pathogen expand the known clinical spectrum of this genus. Traditional MALDI-TOF methods provide only 50% accuracy of species identification in our study. Whole-genome sequencing, *nrdA* sequencing, or multilocus sequence typing can accurately identify 18 species commonly misidentified as *A. xylosoxidans* by conventional methods. Some studies have indicated that while MALDI-TOF is effective for identifying the genus, it is less reliable for species-level identification [12].

Furthermore, WGS enabled the discovery of a novel *Achromobacter* species in patient B, demonstrating the continued evolution and diversification of this genus. This novel species, characterized by a low ANI (<86%) compared to known *Achromobacter* species, highlights the power of WGS in uncovering hidden microbial diversity and expanding our understanding of bacterial taxonomy in the genomic era. We observed the current unclassified *Achromobacter* genomes (sp.) exhibits a greater genomic diversity (Supplementary Figure 3), implying new species within this genus very likely emerge as more genomes are sequenced. A more reliable MALDI-TOF database, including samples from 18 *Achromobacter* species, in combination with whole-genome sequencing and ANI analysis, offers swift and precise identification. For instance, exoproducts from various *Achromobacter* species caused differing levels of IL-6 and IL-8 secretion from epithelial cells, thereby influencing the inflammatory process [13]. Accurate species identification is crucial for proper diagnosis, prognosis prediction, and monitoring of disease trends and spread.

Our study also documented the first reported case of *A. insuavis* bacteraemia in a human. This species, initially identified in the respiratory secretions of cystic fibrosis patients [14], was isolated from the bloodstream of patient F, who had aplastic anaemia and recurrent cellulitis. This finding underscores the potential for *A. insuavis* to cause invasive infections and highlights the importance of considering this species in the differential diagnosis of bacteraemia, particularly in immunocompromised individuals.

*Achromobacter* species harbour a diverse array of intrinsic and acquired resistance mechanisms, including various beta-lactamases [15]. Our analysis revealed a clade-specific distribution of *bla*_OXA_ genes, suggesting that these genes may be lineage-specific and contribute to intrinsic resistance profiles. For instance, all *A. xylosoxidans* isolates carried *bla*_OXA-114-like_ genes, while the novel *Achromobacter* sp. harboured *bla*_OXA-1238_. The identification of *bla*_OXA-364_ in *A. insuavis* and *bla*_AXC-2_ in *A. denitrificans* further expands our knowledge of the beta-lactamase repertoire within this genus.

Bacterial pathogens experience significant adaptive changes in response to the selective forces they face during host colonization and infection, which can evade the effects of antibiotics or the host immune system, potentially by entering a metabolically altered state, or by residing in a protected niche. Genome sequencing provides a sensitive readout of within-host pathogen evolution [16]. In our study, genomic analysis of longitudinal isolates SNPs revealed several missense mutations, including *siaT*, *rapA*, *atzEa1*, and *clpA*. *siaT* is a sialic acid TRAP transporter permease protein. Disrupting the genes that encode *siaT* impairs the growth of *Salmonella enterica* serovar *Typhimurium*, *Clostridioides difficile*, *E. coli*, *S. aureus,* and *Proteus* species [17,18]. Mutations in the *siaT* gene have also been associated with decreased ability of *H. influenzae* to colonize the respiratory tract, underscoring its role in fitness and virulence [19]. The functionality of the *siaT* gene and its encoded transporter is critical for bacterial fitness, particularly in pathogenic bacteria. It influences their ability to acquire nutrients, evade the immune system, adhere to host tissues, and ultimately, their capacity to cause disease.

*rapA* is an RNA polymerase-associated protein. A mutant of an uropathogenic *E. coli* strain affected in the *rapA* gene showed decreased penicillin G resistance. *rapA* gene down-regulated *yhcQ*, which encodes a putative multidrug resistance pump. Mutants in the *rapA* gene also exhibited greater sensitivity to norfloxacin, chloramphenicol, and gentamicin [20]. The change in *Achromobacter* species resistance to beta-lactam antibiotics in patient B may be attributed to the mutation occurring in the *rapA* gene. *AtzE* was likely recruited into the cyanuric acid-mineralizing pathway from an ancestral glutamine transamidosome that required protein–protein interactions to enforce solvent exclusion from the transamidation reaction [21]. *AL523_09115* is a peptide ABC transporter substrate-binding protein. Peptide ABC transporter substrate-binding protein was found to be necessary for *Brucella abortus* virulence [22]. However, there has been no examination of the correlation between antibiotic resistance and the gene involving *AtzE* and *AL523_09115*. Additional research is warranted to explore the further relationship.

Several stress response proteins, including *clpA*, *clpB*, *clpC*, *clpS*, and *clpX*, are members of a family of proteins called the *clp* ATPases, which could protect bacterial pathogens from high temperature and macrophage [23]. The disruption of the genes encoding the *clpP* protease exhibits increased susceptibility to ciprofloxacin. At the same time, mutations affecting the protein *clpS* render resistance against β-lactam antibiotics, such as piperacillin, imipenem, aztreonam, and ceftazidime [24]. Inactivation of *clpA* compromised protease-mediated intrinsic aminoglycoside resistance and weakened *SmeYZ* efflux pump-mediated aminoglycoside resistance of *S. maltophilia* [25]. The change in *A. xylosoxidans* resistance to Amikacin in patient C may be attributed to the mutation occurring in the *clpA* gene.

Considering that antibiotics are frequently employed to treat recurrent infections, often with limited success, it is not surprising that antibiotic resistance is a common issue among many recurrent species. Mutations accumulating in retained genes that regulate the expression of essential metabolic functions, including *rapA* and *clpA* genes, may lead to changes in antimicrobial activity. This longitudinal study has shed light on the potential evolutionary dynamics of how resistance develops *in vivo*, emphasizing the importance of continued surveillance to monitor its dissemination and design more effective clinical strategies to deal with recurrent infections.

This research, however, is subject to some limitations. First, this study collects patients from a single centre. The relatively small sample size may limit the generalization of the results to a larger population. Besides, the lack of incorporation of transcriptomics, proteomics, and metabolomics may restrict the ability to capture pathogen-adaptive trends that converge at the pathway level. Lastly, insufficient validation of mechanisms may limit the capability to assess the impact of adaptations on the host's response. Additional larger-scale research on genomic surveillance of *Achromobacter* species is required to identify emerging variants, monitor the virulence, confirm the mechanisms, and track their dissemination.

## Conclusions

In conclusion, recurrent *Achromobacter* infections are predominantly associated with bloodstream and urinary tract infections, emphasizing the need for vigilant clinical management in these cases. Our study further demonstrates the utility of WGS for accurate species identification, uncovering novel lineages, and tracking within-host evolution of *Achromobacter* species in recurrent infections. The identification of lineage-specific beta-lactamases and the correlation between SNPs and resistance changes provide valuable insights into the molecular mechanisms underlying antibiotic resistance in this emerging pathogen. These findings underscore the importance of WGS-based surveillance to monitor the spread of resistant strains and inform the development of effective strategies to combat *Achromobacter* infections.

## Supplementary Material

Graphical abstract.jpg

Revised Supplement_V2.docx
