# Supplementary figures and images for: Genomic heterogeneity and lineage-specific beta-lactamases in recurrent Achromobacter bloodstream infection patients
Source: Emerg Microbes Infect. 2025 Aug 26;14(1):2547721. doi: 10.1080/22221751.2025.2547721 (PMC12381979; doi:10.1080/22221751.2025.2547721)

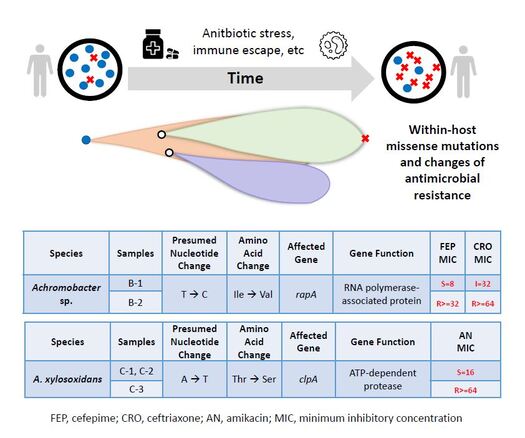

Supplement: Graphical abstract.jpg [file TEMI_A_2547721_SM9394.jpg]
