# Supplementary material for: Genomic heterogeneity and lineage-specific beta-lactamases in recurrent Achromobacter bloodstream infection patients
Source: Emerg Microbes Infect. 2025 Aug 26;14(1):2547721. doi: 10.1080/22221751.2025.2547721 (PMC12381979; doi:10.1080/22221751.2025.2547721)
Supplement: Revised Supplement_V2.docx [file TEMI_A_2547721_SM9393.docx]

**SUPPLEMENTAL MATERIAL**

Genomic Heterogeneity and Lineage-Specific Beta-Lactamases in Recurrent *Achromobacter* Bloodstream Infection Patients

**Supplementary tables 1 to 8**

- Supplementary table 1. *Achromobacter* species infection in human
- Supplementary table 2. Epidemiology of patients with *Achromobacter* species infection
- Supplementary table 3. Demographics and clinical features of patients with recurrent *Achromobacter* bacteremia
- Supplementary Table 4. Multi-locus sequence typing (MLST) of *Achromobacter* isolates
- Supplementary table 5. Antimicrobial susceptibility testing of *Achromobacter* species
- Supplementary Table 6. Assembly status, throughput, and sizes of each isolate
- Supplementary Table 7. RND-type efflux pumps in all 12 isolates
- Supplementary Table 8. Allele frequency shifts at non-synonymous loci

**Supplementary figures 1 to 3**

- Supplementary Figure 1. Multi-locus sequence typing (MLST) of *Achromobacter* isolates
- Supplementary Figure 2. Pairwise ANI of eight pathogenic and two environmental strains
- Supplementary Figure 3. Pairwise ANI of unclassified *Achromobacter* genomes (sp.)

**Supplementary table 1. *Achromobacter* species infection in human**

| Species | Infection sites | Diagnostic tools |
| --- | --- | --- |
| *A. xylosoxidans* | Bacteremia, pneumonia, pharyngitis, CAPD peritonitis, UTI, otitis media | Culture [1], MALDI-TOF [2] |
| *A. denitrificans* | Endocarditis, pneumonia, meningitis, peritonitis, conjunctivitis, osteomyelitis, intra-abdominal abscesses, CAPD peritonitis | MALDI-TOF [3] |
| *A. ruhlandii* | Respiratory tract infection | MALDI-TOF [4] |
| *A. piechaudii* | Otitis, CABSI | *nrdA* sequencing, MALDI-TOF [5] |
| *A. animicus* | Wound infection, respiratory tract infection | MALDI-TOF [6] |
| *A. mucicolens* | Respiratory tract infection | MALDI-TOF [7] |
| *A. pulmonis* | Respiratory tract infection | MALDI-TOF [8] |
| *A. insolitus* | Wound infection, respiratory tract infection | DNA sequencing, MALDI-TOF [9] |
| *A. spanius* | Bacteremia | DNA sequencing, MALDI-TOF [10] |
| *A. deleyi* | Respiratory tract infection | DNA sequencing [11] |
| *A. aegreficans* | Respiratory tract infection | DNA sequencing [12] |
| *A. insuavis* | Respiratory tract infection | DNA sequencing [13] |
| *A. anxifer* | Respiratory tract infection | DNA sequencing [12] |
| *A. dolens* | Respiratory tract infection | MALDI-TOF [14] |
| *A. marplatensis* | Respiratory tract infection | DNA sequencing [15] |

**Supplementary Table 2. Epidemiology of patients with *Achromobacter* species infection**

| Infection Site | Patient Numbers | Patients with Recurrent Infection | Recurrence Percentage (%) | p-value |
| --- | --- | --- | --- | --- |
| Bloodstream Infection | 11 | 6 | 55 | 0.007 |
| Urinary Tract Infection | 18 | 8 | 44 | 0.007 |
| Intra-abdominal Infection | 3 | 1 | 33 | 1 |
| Pneumonia | 23 | 3 | 13 | 0.117 |
| Skin and Soft Tissue Infection | 8 | 1 | 13 | 0.593 |
| Central Nervous System Infection | 1 | 0 | 0 | 1 |
| Sinusitis | 1 | 0 | 0 | 1 |
| Total | 65 | 19 | 29 |  |

**Supplementary Table 3. Demographics and clinical features of patients with recurrent *Achromobacter* bacteremia**

| Patient | Species | Age (years) | Sex  (M/F) | Underlying disease | Suspected infection source | Interval between recurrence | Definitive antibiotics |
| --- | --- | --- | --- | --- | --- | --- | --- |
| A | *A. xylosoxidans* | 60 | F | Rectal cancer | Vesico-rectal fistula infection | 54 days | Piperacillin-tazobactam |
| B | *Achomobacter* sp. | 61 | F | Ovarian cancer | Port A infection | 46 days | Piperacillin-tazobactam |
| C | *A. xylosoxidans* | 39 | F | SLE with lupus nephritis | Central venous catheter infection | 35 days | Piperacillin-tazobactam |
| D | *A. xylosoxidans* | 46 | M | Esophagus cancer | Port A infection | 42 days | Piperacillin-tazobactam |
| E | *A. xylosoxidans* | 63 | M | Oral cancer | Port A infection | 69 days | Piperacillin-tazobactam |
| F | *A. insuavis,*  *A. denitrificans* | 36 | F | Aplastic anemia | Recurrent cellulitis | 79 days | Piperacillin-tazobactam |

**Supplementary Table 4. Multi-locus sequence typing (MLST) of *Achromobacter* isolates**

| Samples | Species | *nusA* | *rpoB* | *eno* | *gltB* | *lepA* | *nuoL* | *nrdA* | *nrdA_765* | Sequence typing |
| --- | --- | --- | --- | --- | --- | --- | --- | --- | --- | --- |
| A-1 | *A. xylosoxidans* | 1 | 26 | 1 | 2 | 59 | 8 | 2 | 36 | ST-315 |
| A-2 | *A. xylosoxidans* | 1 | 26 | 1 | 2 | 193 | 8 | 2 | 36 | ST-591 |
| B-1 | *Achromobacter sp.* | 195 | 135 | 109 | 74 | 133 | 109 | 172 | 381 | ST-594 |
| B-2 | *Achromobacter sp.* | 131 | 135 | 152 | 74 | 133 | 171 | 101 | 382 | ST-592 |
| C-1 | *A. xylosoxidans* | 6 | 26 | 2 | 4 | 195 | 8 | 2 | 36 | ST-595 |
| C-2 | *A. xylosoxidans* | 6 | 26 | 2 | 4 | 59 | 8 | 2 | 36 | ST-207 |
| C-3 | *A. xylosoxidans* | 6 | 26 | 2 | 4 | 59 | 8 | 2 | 36 | ST-207 |
| D-1 | *A. xylosoxidans* | 1 | 26 | 2 | 4 | 62 | 8 | 173 | 140 | ST-593 |
| D-2 | *A. xylosoxidans* | 1 | 26 | 2 | 4 | 62 | 8 | 1 | 140 | ST-448 |
| E-1 | *A. xylosoxidans* | 1 | 46 | 1 | 1 | 59 | 8 | 2 | 125 | ST-315 |
| F-1 | *A. insuavis* | 196 | 48 | 153 | 153 | 194 | 16 | 174 | 380 | ST-596 |
| F-2 | *A. denitrificans* | 44 | 85 | 64 | 50 | 36 | 32 | 45 | 1 | ST-190 |

**Supplementary Table 5. Antimicrobial susceptibility testing of *Achromobacter* species**

| Samples | Species | TGC | GM | AN | SXT | IMP | CAZ | FEP | CRO | CIP | TZP | CZA |
| --- | --- | --- | --- | --- | --- | --- | --- | --- | --- | --- | --- | --- |
| A-1 | *A. xylosoxidans* | S=2 | R>=16 | R>=64 | S<=20 | S=1 | S=2 | I=16 | R>=64 | R>=4 | S<=4 | S=2 |
| A-2 | *A. xylosoxidans* | S=2 | R>=16 | R>=64 | S<=20 | S=1 | S=2 | I=16 | R>=64 | R>=4 | S<=4 | S=2 |
| B-1 | *Achromobacter* sp. | S=2 | R>=16 | R>=64 | S<=20 | S=0.5 | S<=1 | S=8 | I=32 | I=2 | S<=4 | S=2 |
| B-2 | *Achromobacter* sp. | R>=8 | R>=16 | R>=64 | S=40 | S=1 | S=2 | R>=32 | R>=64 | R>=4 | S<=4 | S=2 |
| C-1 | *A. xylosoxidans* | S=1 | R>=16 | S=16 | S<=20 | S=1 | S=4 | R>=32 | R>=64 | I=2 | S<=4 | S=4 |
| C-2 | *A. xylosoxidans* | R>=8 | R>=16 | S=16 | S=80 | S=0.5 | I=16 | R>=32 | R>=64 | R>=4 | S<=4 | R>=8 |
| C-3 | *A. xylosoxidans* | S=2 | R>=16 | R>=64 | S<=20 | S=2 | S=4 | I=16 | R>=64 | I=2 | S<=4 | S=4 |
| D-1 | *A. xylosoxidans* | S=1 | R>=16 | R>=64 | S<=20 | S=1 | S=2 | R>=32 | R>=64 | R>=4 | S<=4 | S=4 |
| D-2 | *A. xylosoxidans* | I=4 | R>=16 | R>=64 | S<=20 | S=1 | S=2 | R>=32 | R>=64 | R>=4 | S<=4 | S=4 |
| E-1 | *A. xylosoxidans* | S=2 | R>=16 | R>=64 | S<=20 | S=1 | S=2 | R>=32 | R>=64 | R>=4 | S<=4 | S=4 |
| F-1 | *A. insuavis* | S=0.5 | R>=16 | R>=64 | S<=20 | S=1 | S=2 | S=8 | R>=64 | S<=1 | S<=4 | S=2 |
| F-2 | *A. denitrificans* | S=0.5 | S=4 | S<=4 | S<=20 | S=1 | S=2 | S=8 | I=32 | S<=1 | S<=4 | S=2 |

TGC, tigecycline; GM, gentamicin; AN, amikacin; SXT, trimethoprim/sulfamethoxazole; IMP, imipenem; CAZ, ceftazidime; FEP, cefepime; CRO, ceftriaxone; CIP, ciprofloxacin; TZP, piperacillin-tazobactam; CZA, ceftazidime-avibactam

**Supplementary Table 6. Assembly status, throughput, and sizes of each isolate**

|  | **Accession** | **Chromosome/Plasmid** | **Throughput (Mbp)** | **Assemby Size (bp)** |
| --- | --- | --- | --- | --- |
| A-1 | CP150720 | Complete Chromosome | 614.9​ | 6433166 |
| A-2 | CP150721 | Complete Chromosome | 579.5​ | 6435971 |
| B-1 | CP150899 | Complete Chromosome | 277.9​ | 6699270 |
| B-2 | CP150897 | Complete Chromosome | 546.0​ | 6701428 |
| C-1 | CP150723 | Complete Chromosome | 683.2​ | 6350934 |
| C-2 | CP150722 | Complete Chromosome | 867.0​ | 6350255 |
| C-3 | CP150898 | Complete Chromosome | 234.7​ | 6348695 |
| D-1 | CP150724 | Complete Chromosome | 264.9​ | 6466641 |
| D-2 | CP150719 | Complete Chromosome | 286.7​ | 6466637 |
| E-1 | CP150718 | Complete Chromosome | 617.3 | 6328122 |
| F-1 | CP150896 | Complete Chromosome | 198.4​ | 6596777 |
| F-2 | CP150725 | Complete Chromosome | 194 | 6741986 |
|  | CP150726 | Complete Plasmid | 194 | 362617 |

**Supplementary Table 7. RND-type efflux pumps in all 12 isolates.**

| **Samples** | **AxyX** | **AxyY** | **OprZ** | **AxyA** | **AxyB** | **AxyM** |
| --- | --- | --- | --- | --- | --- | --- |
| **A-1** | **v** | **v** | **v** | **v** | **v** | **v** |
| **A-2** | **v** | **v** | **v** | **v** | **v** | **v** |
| **B-1** | **v** | **v** | **v** | **v** | **v** | **v** |
| **B-2** | **v** | **v** | **v** | **v** | **v** | **v** |
| **C-1** | **v** | **v** | **v** | **v** | **v** | **v** |
| **C-2** | **v** | **v** | **v** | **v** | **v** | **v** |
| **C-3** | **v** | **v** | **v** | **v** | **v** | **v** |
| **D-1** | **v** | **v** | **v** | **v** | **v** | **v** |
| **D-2** | **v** | **v** | **v** | **v** | **v** | **v** |
| **E-1** | **v** | **v** | **v** | **v** | **v** | **v** |
| **F-1** | **v** | **v** | **v** | **v** | **v** | **v** |
| **F-2** | **v** | **v** | **v** | **v** | **v** | **v** |

**Supplementary Table 8**. Allele frequency shifts at non-synonymous SNP loci between recurrent samples. Percentages represent the observed allele frequency of each nucleotide (A, C, G, T) per sample. The major alleles are bolded. *P* values were computed using a Dirichlet–multinomial likelihood ratio test.

| Genes | SNP locus | A | | C | G | | T | *P* value |
| --- | --- | --- | --- | --- | --- | --- | --- | --- |
| *siaT* | 2418800 | 0% | 1% | | 0% | **99%** | | 0.000205 |
|  |  | **92%** | 0% | | 6% | 2% | |  |
| *rapA* | 3605691 | 7% | 33% | | 0% | **60%** | | 0.001135 |
|  |  | 6% | **56%** | | 9% | 29% | |  |
| *atzEa1* | 4409525 | 0% | 9% | | 0% | **91%** | | 0.002958 |
|  |  | 8% | **50%** | | 0% | 42% | |  |
| *AL523_09115* | 4557476 | 26% | 0% | | **72%** | 2% | | 0.002855 |
|  |  | **48%** | 3% | | 41% | 8% | |  |
| *clpA* | 3213646 | **99%** | 0% | | 1% | 0% | | 0.004284 |
|  |  | **98%** | 1% | | 1% | 0% | |  |
|  |  | 3% | 0% | | 0% | **97%** | |  |
| gbpRa1 | 357552 | 3% | 0% | | **50%** | 47% | | 0.10805 |
|  |  | 0% | 1% | | 46% | **53%** | |  |
| lhgD | 2532145 | 31% | 0% | | **69%** | 0% | | 0.156797 |
|  |  | **53%** | 0% | | 47% | 0% | |  |
| smvA | 5162109 | 0% | **73%** | | 5% | 23% | | 0.02477 |
|  |  | 0% | **49%** | | 2% | **49%** | |  |
| yfcGa1 | 5618153 | 0% | 0% | | 36% | **64%** | | 0.017192 |
|  |  | 0% | 8% | | **47%** | 44% | |  |

**Supplementary Figure 1. Whole-genome phylogeny of the 12 *Achromobacter* isolates using *A. piechaudii* LMG 1861 as the outgroup**


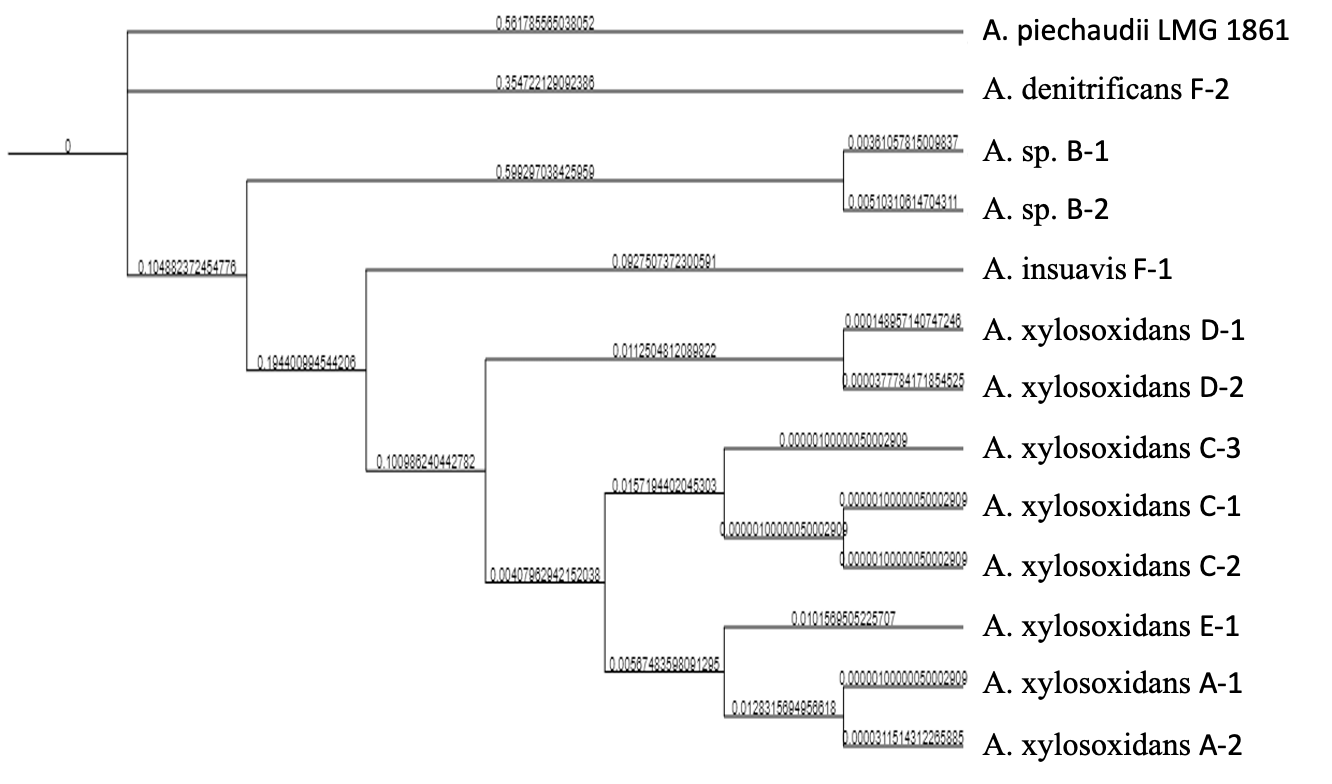


**Supplementary Figure 2. Pairwise ANI of eight pathogenic (Blood: *A. xylosoxidans*​ A-1, *A. xylosoxidans*​ A-2, *A. xylosoxidans* D-1, *A. xylosoxidans* D-2, Ear discharge (otitis): RM8376, NCTC1087; sputum: FDAARGOS_1091; Urine: 2021CK-01139) and two environmental strains (H1_3_1, YYS002)**


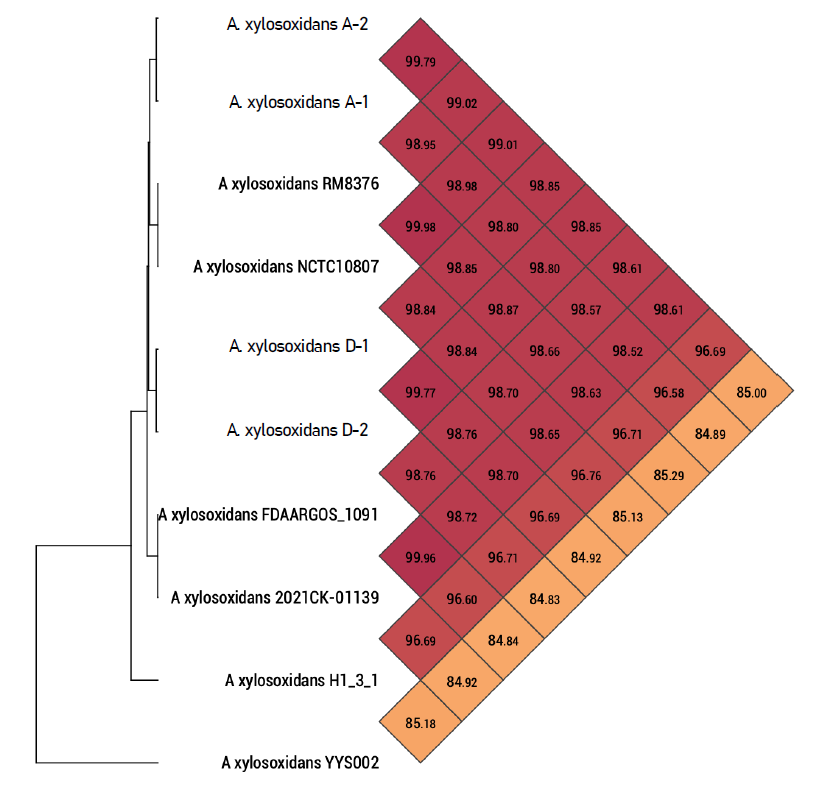


**Supplementary Figure 3. Pairwise ANI of unclassified *Achromobacter* genomes (sp.)**
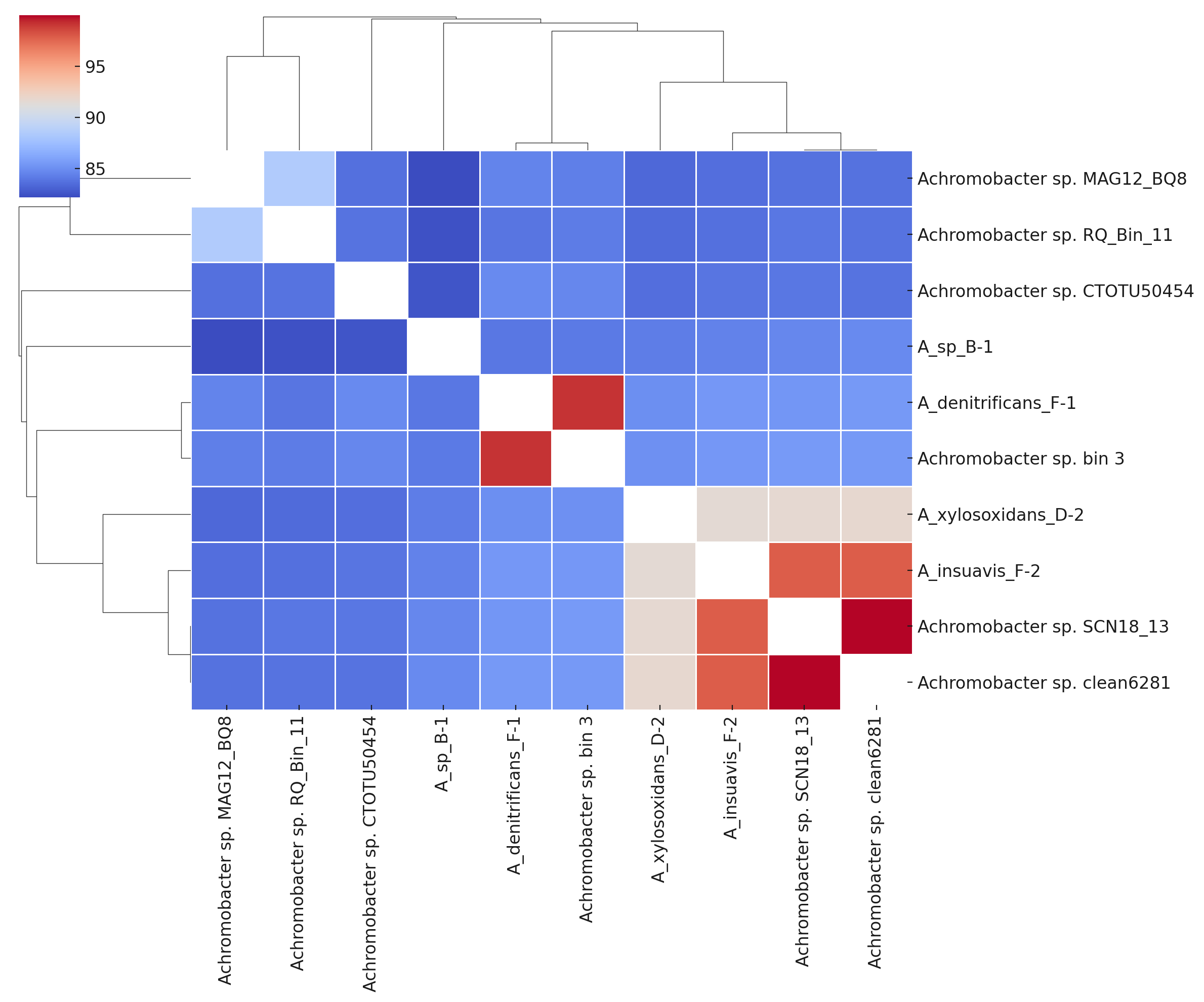


**Reference**

1. Igra-Siegman Y, Chmel H, Cobbs C. Clinical and laboratory characteristics of Achromobacter xylosoxidans infection. *J Clin Microbiol.* 1980;11(2):141-145.

2. Pérez Barragán E, Sandino Pérez J, Corbella L, Orellana MA, Fernández-Ruiz M. Achromobacter xylosoxidans bacteremia: clinical and microbiological features in a 10-year case series. *Rev Esp Quimioter.* 2018;31(3):268-273.

3. Marko DC, Saffert RT, Cunningham SA, et al. Evaluation of the Bruker Biotyper and Vitek MS matrix-assisted laser desorption ionization-time of flight mass spectrometry systems for identification of nonfermenting gram-negative bacilli isolated from cultures from cystic fibrosis patients. *J Clin Microbiol.* 2012;50(6):2034-2039.

4. Garrigos T, Dollat M, Magallon A, et al. Distribution of Achromobacter Species in 12 French Cystic Fibrosis Centers in 2020 by a Retrospective MALDI-TOF MS Spectrum Analysis. *J Clin Microbiol.* 2022;60(6):e0242221.

5. Garrigos T, Neuwirth C, Chapuis A, Bador J, Amoureux L; Collaborators. Development of a database for the rapid and accurate routine identification of Achromobacter species by matrix-assisted laser desorption/ionization-time-of-flight mass spectrometry (MALDI-TOF MS). *Clin Microbiol Infect.* 2021;27(1):126.e1-126.e5.

6. Moremi N, Claus H, Hingi M, Vogel U, Mshana SE. Multidrug-resistant Achromobacter animicus causing wound infection in a street child in Mwanza, Tanzania. *Diagn Microbiol Infect Dis.* 2017;88(1):58-61.

7. Al-Asadi SA, Al-Kahachi RES, Alwattar WMA, Bootwala J, Sabbah MA. Genomic Insights into Achromobacter mucicolens IA Antibiotic Resistance. *Microbiol Spectr.* 2022;10(2):e0191621.

8. Veschetti L, Boaretti M, Saitta GM, et al. Achromobacter spp. prevalence and adaptation in cystic fibrosis lung infection. *Microbiol Res.* 2022;263:127140.

9. Li G, Zhang T, Yang L, et al. Complete genome sequence of Achromobacter insolitus type strain LMG 6003T, a pathogen isolated from leg wound. *Pathog Dis.* 2017;75(4):10.1093/femspd/ftx037.

10. Li G, Yang L, Zhang T, et al. Complete genome sequence of Achromobacter spanius type strain DSM 23806T, a pathogen isolated from human blood. *J Glob Antimicrob Resist.* 2018;14:1-3.

11. BacDive. Achromobacter deleyi DSM 26072 is a mesophilic human pathogen that was isolated from human sputum, cystic fibrosis patient. [cited 2024 March 27] https://bacdive.dsmz.de/strain/303

12. Vandamme P, Moore ER, Cnockaert M, et al. Classification of Achromobacter genogroups 2, 5, 7 and 14 as Achromobacter insuavis sp. nov., Achromobacter aegrifaciens sp. nov., Achromobacter anxifer sp. nov. and Achromobacter dolens sp. nov., respectively. *Syst Appl Microbiol.* 2013;36(7):474-482.

13. Nielsen SM, Nørskov-Lauritsen N, Bjarnsholt T, Meyer RL. Achromobacter Species Isolated from Cystic Fibrosis Patients Reveal Distinctly Different Biofilm Morphotypes. *Microorganisms.* 2016 Sep 14;4(3):33.

14. Veschetti L, Sandri A, Patuzzo C, Melotti P, Malerba G, Lleo MM. Genomic characterization of Achromobacter species isolates from chronic and occasional lung infection in cystic fibrosis patients. *Microb Genom.* 2021;7(7):000606. doi:10.1099/mgen.0.000606

15. BacDive. Achromobacter marplatensis AU 14580 is an aerobe, mesophilic, Gram-negative bacterium that was isolated from non-cystic fibrosis patient, sputum. [cited 2024 March 27] https://bacdive.dsmz.de/strain/130145
